# Problematic Drug Use Among Outpatients With Schizophrenia and Related Psychoses

**DOI:** 10.3389/fpsyt.2021.762988

**Published:** 2021-10-22

**Authors:** Sherilyn Chang, Anitha Jeyagurunathan, Jue Hua Lau, Saleha Shafie, Ellaisha Samari, Laxman Cetty, Yee Ming Mok, Swapna Verma, Mythily Subramaniam

**Affiliations:** ^1^Research Division, Institute of Mental Health, Singapore, Singapore; ^2^Department of Mood and Anxiety, Institute of Mental Health, Singapore, Singapore; ^3^Department of Psychosis, Institute of Mental Health, Singapore, Singapore

**Keywords:** drug misuse, drug use, schizophrenia, DAST, substance misuse

## Abstract

**Background:** Problematic drug use is common among psychiatric patients and is linked with poorer course and outcomes of illness. The aim of this study is to assess the prevalence of problematic drug use, and to explore its sociodemographic correlates and associations with health behaviors and outcomes among outpatients with schizophrenia and related psychoses in Singapore.

**Methods:** Data from 397 individuals who were aged 21–65 years and were seeking treatment for schizophrenia and related psychoses in the outpatient clinics of a tertiary psychiatric hospital were analyzed. The Drug Abuse Screening Test (DAST-10) was used to assess problematic drug use. Information on sociodemographics, smoking status, alcohol use, symptoms severity and quality of life were collected. Multivariable logistic regressions were conducted to explore correlates and associations of problematic drug use.

**Results:** The prevalence of problematic drug use was 5.8% (*n* = 23) in the sample, and 10.6% (*n* = 42) of the participants reported having problematic drug use and/or problematic alcohol use. More males than females reported having problematic drug use (*p* = 0.021), and also problematic drug and/or alcohol use (*p* = 0.004). Significant associations were observed between problematic drug use and smokers with nicotine dependence, and with physical health domain of quality of life. Individuals with greater symptom severity were approximately twice as likely to have problematic drug use and/or alcohol use.

**Conclusion:** While the prevalence of problematic drug use in this sample population is relatively lower compared to other countries, there is a considerable number who might be at risk. Routine screening and close monitoring of drug use is recommended as part of psychiatric assessment, particularly among males and patients with nicotine dependence.

## Introduction

Drug use is common among individuals with schizophrenia. The rate of comorbid substance use disorder in persons with schizophrenia ranges widely between 10 and 70%, with variations largely attributed to methodological differences such as study population and variability in definitions of substance use (1). Data from the US Epidemiological Catchment Area Study showed that 47.0% of individuals with a lifetime diagnosis of schizophrenia or schizophreniform disorder met the criteria for any substance use (abuse and dependence), 27.5% met the criteria for any drug (other than alcohol) disorder, and 33.7% met criteria for an alcohol disorder (2). The odds of having these comorbid conditions were three to six times higher among those with schizophrenia as compared to the general population. A case-control study similarly reported higher number of patients with schizophrenia who had problem use of drugs and alcohol as compared to matched controls from the general population (3). More recently, a meta-analysis established a lifetime rate of cannabis use disorder at 27.1% among clinical samples of patients with schizophrenia (4). Drug use contributes to increased risk of psychosis (5) and while for some individuals such substance-induced psychosis could be a transient state, the clinical conditions could persist, mimicking the positive and negative symptoms observed in schizophrenia (6). The transition from substance-induced psychosis to primary psychotic disorder has been reported in the literature (7, 8).

Problematic use of drugs as discussed in this article generally refers to the use of drugs that resulted in physical, psychological, social and legal problems [for definitions, see Refs. (9) and (10)]. It has been linked with poorer course and outcomes for patients with schizophrenia. Drug abuse was a significant predictor of self-harm and suicide among those with schizophrenia (11, 12), and individuals with a dual diagnosis of substance use disorder and schizophrenia reported higher positive and negative symptoms, and were more likely to be medication non-compliant and depressed compared to those with a single diagnosis of schizophrenia (13). A longitudinal study among patients with recent onset of psychosis found that those with persistent use of cannabis had more positive symptoms and a continuous illness (i.e., no remission longer than 6 months) at follow-up (14). Furthermore, substance abuse in patients with schizophrenia was associated with increased rates of hospitalizations, incarceration and use of emergency services, as well as higher treatment expenditure (15, 16).

There is some evidence in the literature showing the effectiveness of treating drug-related problems among patients with a dual diagnosis of schizophrenia and substance use disorder (17–19). This included improvements in psychiatric outcomes in areas of global functioning, positive symptoms and quality of life, and in substance-related outcomes in terms of relapse and days abstinent. However, some studies have reported limited evidence on the effectiveness of psychosocial interventions (20, 21), and the absence of high-quality randomized controlled trials precluded definitive conclusions (22). Nonetheless, given the negative outcomes of substance use disorder in patients with schizophrenia and the potential improvement in outcomes with interventions, it remains important to identify the extent of problematic drug use in this population.

Singapore is a multi-ethnic city-state located in Southeast Asia with a resident population of 4.04 million (23). While the lifetime prevalence of schizophrenia and psychotic disorder in Singapore was established at 2.3% in the general population (24), little is known about the prevalence of problematic drug use among those with the condition. In the general population, the total number of individuals arrested for illicit drug use was 3,014 in 2020, a 15% drop from the previous year, and Methamphetamine remained the most commonly used drug (25). To date, only one study has been conducted in Singapore among patients with schizophrenia to examine substance abuse [alcohol and other substances; (26)], and two studies have examined hazardous alcohol use among patients with first episode psychosis, and among a mixed sample of outpatients with schizophrenia and depressive disorders (27, 28). Given the paucity of research particularly on drug use, this study was thus conducted with the aim to (i) establish the prevalence of problematic drug use in a clinical sample of patients with schizophrenia and related psychoses, (ii) identify sociodemographic correlates of problematic drug use, and (iii) explore associations between problematic drug use and smoking status, symptoms severity as well as quality of life.

## Methods

### Study Design and Sample

The study utilized data collected from a cross-sectional study conducted at the Institute of Mental Health (IMH), a tertiary psychiatric hospital in Singapore. Recruitment of participants took place between October 2019 and March 2021, with a temporary suspension between April 2020 and June 2020 due to a nationwide lockdown in response to the COVID-19 pandemic. Face-to-face recruitment and data collection resumed after which, subsequent participants were given the option to complete the study online should they prefer. Participants were outpatients seeking treatment at IMH and were recruited through convenience sampling following referrals from clinicians and other mental healthcare professionals (e.g., case managers, researchers). They were invited to participate in the study if the following inclusion criteria were met: (1) Singapore citizens or permanent residents aged 21–65 years; (2) clinically diagnosed with schizophrenia or having related psychoses, as determined by a psychiatrist following the Diagnostic and Statistical Manual of Mental Disorders-IV (29) criteria; and (3) able to read and understand English. Prior to data collection, written informed consent was obtained from participants who were recruited face-to-face, while online electronic consent was taken from those who completed the study online. The study was approved by the relevant institutional ethics committee (National Healthcare Group Domain Specific Review Board).

### Measures

#### Drug Abuse Screening Test (DAST)

The DAST-10 is a brief self-reported instrument to assess misuse of drugs, excluding alcohol and tobacco, in the past year (30). Items were given a score of 1 for a “yes” response (except for Item 3 which is reversed scored, where “no” was given a score of 1) to questions related to maladaptive drug use behavior and its consequences (e.g., “Have you used drugs other than those required for medical reasons?”; “Have you neglected your family because of your use of drugs?”), and a total score was obtained by summing items across the scale. The total score can be interpreted as the degree of problematic drug use, with categories reflecting “none” (0), “low” (1–2), “moderate” (3–5), “substantial” (6–8) and “severe” (9–10) level of problems. For this study, a cut-off score of ≥3 was used to index significant problematic drug use as it has demonstrated high sensitivity and specificity in validation studies among psychiatric population (31, 32). The scale showed good internal consistency in this study sample with a Cronbach's alpha of 0.73.

#### Fagerstrom Test for Nicotine Dependence (FTND)

Information on smoking behavior was collected by first asking participants if they were current smokers. Those who responded “yes” proceeded to complete the 6-item FTND which assessed physiological dependence on tobacco smoking (33). Items in the scale were summed to yield a total score ranging from 0 to 10. Following studies conducted in the local general population and among psychiatric patients, a cut-off score of ≥5 was used to indicate nicotine dependence (34, 35). Participants were then classified as either “non-smokers,” “smokers without nicotine dependence” or “smokers with nicotine dependence”.

#### Cut-Annoyed-Guilty-Eye (CAGE)

The CAGE questionnaire uses four items to assess self-reported problems related to alcohol use (36). These items were prefaced by a screening question that asked “Was there ever a period in your life when you drank at least 12 drinks in a year?” Participants who answered “yes” to the screening question completed the four items in the CAGE tool regarding their drinking habits. Endorsing two or more items in the CAGE tool was indicative of problematic alcohol use in this study. Those who answered “no” to the screening or indicated that they had never drunk alcohol were directed to skip the CAGE questionnaire. Participants were thus classified as either “non-drinkers,” “drinkers without problems” or “drinkers with problematic alcohol use.” The tool has been validated (37) and has been used to examine alcohol consumption in the local population (38). A moderate internal consistency of this scale was obtained for this sample (α = 0.69).

#### Symptoms Checklist-90-Revised (SCL-90-R)

The SCL-90-R is a widely used instrument that provides a measure of psychiatric distress and severity of psychopathology symptoms (39). The checklist consists of 90 items and respondents were asked to rate how much they were bothered by the symptoms in the past week using a five-point Likert scale from 0 = Not at all to 4 = Extremely. Total scores for nine primary symptom dimensions and three global measures of psychological distress can be obtained from the scale. The Global Severity Index (GSI) was calculated by taking the average of all items, with higher scores indicating greater distress and symptom severity. Internal consistency of the scale was high for the study sample (α = 0.99).

#### World Health Organization Quality of Life-BREF (WHOQOL-BREF)

This 26-item instrument assesses subjective evaluation of personal health and well-being over the past 2 weeks using a 5-point Likert scale (40). It covers four domains: physical health, psychological health, social relationships, and environment. Domain scores were calculated by taking the mean score of items within each domain and multiplied by 4 to transform the value to a 4–20 scale; higher scores reflect greater satisfaction and higher QoL in the domain. The scale has previously been validated in Singapore and has obtained sound psychometric properties (41, 42).

Sociodemographic information including age, gender, ethnicity, highest educational attainment, marital status and monthly personal income [in Singapore dollars (SGD)] were collected.

### Statistical Analysis

Descriptive analyses were performed to describe the participant profile. Mean and standard deviations were calculated for continuous variables, and frequencies and percentages were computed for categorical variables. In order to produce more reliable estimates, ethnicity was reclassified as Chinese vs. non-Chinese, and the “primary and below” and “secondary” categories for educational attainment were regrouped as a single category “secondary and below” for subsequent analyses. Independent *T*-test, Chi-square test and Fisher's exact test were conducted to explore sociodemographic correlates of problematic drug use at a bivariate level. Associations between problematic drug use and smoking status, symptoms severity and quality of life were assessed using multivariable logistic regression. Problematic drug use was treated as outcome variable and symptom severity, smoking status, and quality of life (all four domains) as independent variables. To improve stability of the model and its estimates, sociodemographic variables found to be significant in bivariate analyses were included in the regression model. Noting the relatively low prevalence of problematic drug use in this study sample, the same set of analyses were conducted to further examine correlates and associations with “problematic drug use and/or alcohol use” as the outcome variable. All statistical analyses were performed using IBM SPSS Statistics for Windows, version 23 (IBM Corp., Armonk, N.Y., USA), except for Fisher's exact test which was conducted using Stata version 17, and statistical significance was set at *p*-value <0.05.

## Results

### Sample Characteristics

A total of 400 participants were recruited for the study. However, three cases were excluded from analysis for the following reasons: (i) one person being enrolled twice in the study, (ii) one participant requested to withdraw from study, and (iii) one participant was above the age limit of the study. The final study sample consisted of 397 participants.

The mean age of participants was 36.2 years (SD = 10.9; Table 1). There was an approximately equal number of male and female participants and the majority of them were Chinese (74.8%) and single (80.6%). The mean SCL-90-R Global Severity Index was 0.94 (SD = 0.9).

**Table 1:** Profile of study sample (*n* = 397).

		***n***	**%**
Age (Mean, SD)		36.2, 10.9	
Gender	Female	196	49.4
	Male	201	50.6
Ethnicity^a^	Chinese	297	74.8
	Malay	51	12.8
	Indian	38	9.6
	Others	11	2.8
Educational attainment	Primary and below^b^	18	4.5
	Secondary^b^	120	30.2
	GCE “A” Level/Diploma/Vocational training	182	45.8
	Degree and above	77	19.4
Marital status	Single	320	80.6
	Married	47	11.8
	Separated/Divorced/Widowed	30	7.6
Monthly personal income^c^	No income	114	30.1
	Below S$2,000	202	53.3
	S$2,000–S$3,999	48	12.7
	S$4,000 and above	15	4.0
Problematic drug use^d^	None (0)	353	89.6
	Low (1–2)	18	4.6
	Moderate (3–5)	17	4.3
	Substantial (6–8)	5	1.3
	Severe (9–10)	1	0.3
Problematic alcohol use	Non-drinkers	347	87.4
	Drinkers without problems	29	7.3
	Drinkers with problematic alcohol use	21	5.3
Smoking status^c^	Non-smokers	307	78.3
	Smokers without nicotine dependence	31	7.9
	Smokers with nicotine dependence	54	13.8

a *Ethnicity was reclassified as Chinese vs. Non-Chinese for subsequent analyses*.

b *These two categories were regrouped as a single category for subsequent analyses*.

c *Remaining responses were “Refuse/Don't Know” and were treated as missing data. Valid percentages are presented*.

d *Numbers in bracket indicate respective range of DAST score. Valid percentages are shown after accounting for 3 missing data cases*.

### Prevalence of Problematic Drug Use and Its Correlates

The prevalence of problematic drug use (DAST-10 score ≥3) in this sample was 5.8% (*n* = 23; Table 1). Though not meeting the DAST-10 cut-off score, 4.6% of the participants had a low degree of problematic drug use. 5.3% (*n* = 21) of the study sample reported having drinking problems, and the prevalence of problematic drug use and/or alcohol use was 10.6% (*n* = 42).

Gender was the only significant correlate found to be associated with problematic drug use at bivariate level (males 8.5% vs. females 3.1%, *p* = 0.021; Table 2). There were also significantly more males than females (15.1 vs. 6.2%) who reported having problematic drug use and/or alcohol use (*p* = 0.004).

**Table 2:** Sociodemographic correlates of problematic drug use and/or alcohol use.

		**With problematic drug use (DAST-10 ≥3)**			**With problematic drug and/or alcohol use**		
		**Yes**	**No**		**Yes**	**No**	
		***n***	***n***	***p*-value**	***n***	***n***	***p*-value**
Age (Mean)		35.7	36.1	0.838	36.2	36.1	0.933
Gender	Female	6 (3.1)	189 (96.9)	**0.021**	12 (6.2)	183 (93.8)	**0.004**
	Male	17 (8.5)	182 (91.5)		30 (15.1)	169 (84.9)	
Ethnicity	Chinese	17 (5.8)	278 (94.2)	0.913	29 (9.8)	266 (90.2)	0.357
	Non-Chinese	6 (6.1)	93 (93.9)		13 (13.1)	86 (86.9)	
Educational attainment	Secondary and below	8 (5.9)	128 (94.1)	0.753	16 (11.8)	120 (88.2)	0.870
	GCE “A” Level/ Diploma/Vocational training	12 (6.6)	169 (93.4)		18 (9.9)	163 (90.1)	
	Degree and above	3 (3.9)	74 (96.1)		8 (10.4)	69 (89.6)	
Marital status	Single	17 (5.4)	300 (94.6)	0.465	31 (9.8)	286 (90.2)	0.198
	Married	3 (6.4)	44 (93.6)		5 (10.6)	42 (89.4)	
	Separated/Divorced/Widowed	3 (10.0)	27 (90.0)		6 (20.0)	24 (80.0)	
Monthly personal income	No income	9 (8.1)	102 (91.9)	0.125	15 (13.5)	96 (86.5)	0.075
	Below S$2,000	8 (4.0)	194 (96.0)		17 (8.4)	185 (91.6)	
	S$2,000–S$3,999	1 (2.1)	47 (97.9)		3 (6.3)	45 (93.8)	
	S$4,000 and above	2 (13.3)	13 (86.7)		4 (26.7)	11 (73.3)	

*Numbers in bracket represent row percentages. p-value obtained from T-test for continuous variable and Chi-square or Fisher's exact test for categorical variables. Significant values (p < 0.05) are bold*.

### Associations With Smoking Status, Symptoms Severity and Quality of Life

Problematic drug use was significantly associated with smoking status. Compared to non-smokers, smokers with nicotine dependence were ~5 times as likely to have problematic use of drugs (OR = 4.79, 95% CI: 1.57–14.61; Table 3). A higher score in physical health domain of quality of life was associated with lower odds of problematic drug use (OR = 0.65, 95% CI: 0.48–0.88), while a higher score in the environmental domain was associated with higher odds of problematic drug use (OR = 1.29, 95% CI: 1.01–1.65).

**Table 3:** Associations with smoking status, symptoms severity, and quality of life.

	**Problematic drug use**			**Problematic drug and/or alcohol use**		
		**95% Confidence interval**			**95% Confidence interval**	
	**OR^a^**	**Lower bound**	**Upper bound**	**OR^a^**	**Lower bound**	**Upper bound**
**Smoking status**						
Non-smokers	Reference			Reference		
Smokers without nicotine dependence	4.37	0.98	19.41	2.57	0.69	9.54
Smokers with nicotine dependence	**4.79**	1.57	14.61	**3.81**	1.51	9.61
**Symptom severity**						
Global Severity Index	1.36	0.74	2.51	**1.89**	1.16	3.09
**Quality of life**						
Physical health	**0.65**	0.48	0.88	**0.71**	0.57	0.90
Psychological health	1.14	0.88	1.47	1.11	0.91	1.35
Social relationship	0.99	0.82	1.20	0.95	0.82	1.10
Environment	**1.29**	1.01	1.65	**1.26**	1.03	1.53

a *Odds ratio derived from multivariable logistic regression controlled for gender. Results in bold indicate significant findings (p < 0.05)*.

Similar associations were observed between problematic drug use and/or alcohol use and smoking status as well as quality of life. Smokers with nicotine dependence were ~4 times as likely to have problematic drug use and/or alcohol use than non-smokers (OR = 3.81, 95% CI: 1.51–9.61). Higher score in the physical health domain of quality of life was associated with lower odds of problematic drug use and/or alcohol use (OR = 0.71, 95% CI: 0.57–0.90), and higher score in the environmental domain was associated with higher odds of problematic drug use and/or alcohol use (OR = 1.26, 95% CI: 1.03–1.53). Additionally, greater symptom severity was associated with increased odds of problematic drug use and/or alcohol use (OR = 1.89, 95% CI: 1.16–3.09).

## Discussion

The present study aimed to establish the prevalence of problematic drug use among patients with schizophrenia and related psychoses. It was found that 5.8% of patients surveyed in this study reported misusing drugs in the past 1 year, and 10.6% had problems with drug use and/or alcohol use. Notwithstanding the differences in study methodologies, our finding suggests that problematic drug use is less common among patients with schizophrenia in Singapore as compared to other countries (3, 4).

In general, the use of illicit drugs is lower in Singapore than most other countries, and this largely reflects Singapore's zero tolerance approach to drugs (43) and the success of anti-drug movement in the country. The Central Narcotics Bureau is the primary drug enforcement agency in Singapore and has four main strategies in place to keep the nation drug-free: preventive drug education, rigorous enforcement, treatment and rehabilitation, and aftercare and continued rehabilitation. These efforts are supported by other agencies including the Singapore Anti-Narcotics Association and the Yellow Ribbon Singapore. In the recent years, a rehabilitative and integrative approach toward individuals with drug related offenses has been embraced, and through community-based programs where efforts in early reintegration of these individuals into the community are made (44). Nonetheless, some challenges may remain in drug control and treatment strategies such as a need to focus on familial interventions and more research in the area of drug addiction (45, 46).

Results from the study revealed a considerable number of patients with schizophrenia who may be at risk of significant problematic drug use. Though not meeting the threshold in this study to be classified as having significant problematic drug use, 4.6% of those surveyed had a low degree of problematic drug use (i.e., DAST-10 score: 1–2). It remains that this group should be closely monitored for their drug use and brief counseling may be recommended for them (47). Given that several reasons have been identified for illicit drug use among individuals with psychotic disorders, including social reasons and as coping mechanism for symptoms and side-effects of medications (48, 49), future studies can aim to explore drug use motivations within this at-risk group to effectively intervene at an early stage. Furthermore, understanding the frequency and types of drugs used would be essential. In the local context, the study by Verma et al. (26) found that benzodiazepine was one of the frequently abused substances among patients with schizophrenia and the authors called for caution when prescribing these medications.

Consistent with studies in the literature which identified gender as a significant correlate of substance use (50–53), the current study found that more males than females had problematic drug use, and also problematic drug and/or alcohol use. Data from national statistics have shown that there were consistently more males than females who used illicit drugs in Singapore (54). Such a gender disparity could reflect differences in opportunity to use drug rather than vulnerability; males were found to have greater opportunities to use drug, but when once presented with the opportunity, females were as likely as males to initiate drug use (55). While the severity of substance use between both genders might be similar (56), females might be more susceptible to the negative effects of drugs use (57, 58) and also more likely to relapse following abstinence (59). Additionally, males and females with serious mental illness were found to differ in drug use behavior including ways to drugs access and reasons for drug use. Women were more likely to have drugs given to them by a significant other, and to report using drugs to test their ability to control drug use (60). In terms of hazardous alcohol use, several biological risk factors (e.g., genetic risks and gender differences in physiological effects of alcohol) and psychosocial risk factors (e.g., relating to social norms and general roles) have been proposed to account for the gender differences (61). These findings collectively suggest that effective treatment plans targeting problematic drug and alcohol use among patients with schizophrenia and related psychoses would need to consider such gender differences.

In line with studies that established the link between smoking and drug and alcohol use in patients with schizophrenia (62, 63) and also in the general population (64, 65), this study found that smoking behavior in patients with schizophrenia is significantly associated with problematic drug use. Smokers with nicotine dependence were five times more likely to have problematic drug use as compared to non-smokers, and four times more likely to have problematic drug use and/or alcohol use. These findings provided additional evidence of the close association between substance misuse and schizophrenia. Many hypotheses have been proposed to explain for this phenomenon, including common underlying genetic factors that predispose individuals to substance use behavior and schizophrenia, and also the self-medication hypothesis which suggests that patients use substance to cope with their psychiatric symptoms and the side-effects of medications (66). There also exist evidence for the gateway hypothesis which posits that smoking serves as a gateway substance to illicit drug use (67, 68). Given the high co-occurrence of problematic drug use and alcohol use along with nicotine dependence, it is recommended that routine assessments of nicotine use be conducted among patients with schizophrenia and related psychoses to closely evaluate for potential problems with drug use and alcohol use.

This study found that patients with greater symptoms severity were more likely to have problematic drug use and/or alcohol use. A study by Spencer et al. (69) similarly demonstrated symptoms severity as a significant predictor of cannabis or alcohol use among individuals with psychotic disorder, and this was mediated by their motives for using substances; worse symptoms resulted in stronger motives which in turn lead to stronger psychological dependence on substances. It may also be plausible that the problematic use of drug and alcohol contributed to greater symptoms severity. A study among older adults with schizophrenia found that higher levels of alcohol consumption was associated with higher levels of general psychopathology, and among them individuals with comorbid alcohol abuse had more severe negative symptoms and general psychopathology (70). Similarly, in a longitudinal study among persons with psychotic disorder, a reduction in the quantity of alcohol consumed predicted reduction in depressive symptoms, though not with anxiety nor psychotic symptoms (71). However, contradictory findings have been reported in the literature where some patients experienced symptom reduction (decreased anxiety and depression) following alcohol and cannabis use (72), which may in part be due to differences in the types of substance examined and study methodologies.

A significant negative association was observed between physical health domain of quality of life and problematic drug use and/or alcohol use. It may be plausible that patients who perceived poor health from the physical side effects of anti-psychotic medications used illicit drugs or alcohol to relieve such negative experiences (49, 73). It is equally plausible that poor health could be directly attributed to the consequences of misusing drugs (74). Prior studies have similarly reported poorer quality of life among patients with schizophrenia who were current substance users (75) and among those with current stimulant drug use (76). However, contrary to these findings, the study by Herman (77) found that inpatients with comorbid schizophrenia and substance abuse disorder reported better quality of life than those non-substance abusing inpatients. The author attributed this disparity to group differences in terms of lower levels of psychopathology and better executive functioning in the former group. Future studies may look into exploring these clinical variables in understanding the associations of problematic drug use in patients with schizophrenia and related psychoses.

There are a few limitations of this study to be considered when interpreting the results. Firstly, participant's self-reported data was used and thus the extent of problematic drug use could be underestimated due to social desirability bias. However, measures were taken to reduce such bias whereby participants were reassured regarding data confidentiality and were given the privacy to complete the questionnaire on their own. Next, the data collection period overlapped with the COVID-19 pandemic and the demand and supply of illicit drugs could have been affected due to movement restrictions across country borders. This might have influenced the availability and thus frequency of drug use among the study participants. The cross-sectional design of the study limits the ability to draw conclusions on the causal effects of associations examined. Lastly, as participants recruited in this study were outpatients, findings may not be generalizable to inpatient setting where clinical profile of patients differs (e.g., severity of psychotic symptoms, higher comorbidity with substance use) and is likely to influence the prevalence and associations established in this study.

Despite its limitations, this study in many ways contributed to extant literature on problematic drug use among patients with schizophrenia and related psychoses. Most studies have explored substance misuse in psychiatric population and relatively few have focused solely on non-alcohol misuse. Having a clearer differentiation and studying a distinct category of substance misuse allows researchers and healthcare professionals to disentangle correlates and effects of different substances. This study has examined both problematic drug use, and also problematic drug and/or alcohol use among patients with schizophrenia and related psychoses. Given the paucity of related research conducted in Singapore, results from this study can provide valuable insights into drug misuse in the local context and inform healthcare professionals when developing care plans tailored to patients' needs.

## Data Availability Statement

Data may be available upon reasonable request and subjected to approval by the institutional review board (IRB). This is a requirement mandated for this research study by our IRB and funders. Requests to access the dataset should be directed to the senior author, Mythily Subramaniam, mythily@imh.com.sg.

## Ethics Statement

The studies involving human participants were reviewed and approved by National Healthcare Group Domain Specific Review Board. The patients/participants provided their written informed consent to participate in this study.

## Author Contributions

AJ, YM, SV, and MS were involved in the design and conception of the study. SC, AJ, JL, SS, ES, and LC were involved in participant recruitment and data collection. SC undertook the data analysis with assistance from JL. SC drafted the manuscript with supervision from AJ and MS. All authors contributed to the article and approved the submitted version.

## Funding

The study was funded through the Singapore Ministry of Health's National Medical Research Council under the Centre Grant Programme (Grant No: NMRC/CG/M002/2017_IMH).

## Conflict of Interest

The authors declare that the research was conducted in the absence of any commercial or financial relationships that could be construed as a potential conflict of interest.

## Publisher's Note

All claims expressed in this article are solely those of the authors and do not necessarily represent those of their affiliated organizations, or those of the publisher, the editors and the reviewers. Any product that may be evaluated in this article, or claim that may be made by its manufacturer, is not guaranteed or endorsed by the publisher.
